# Supplementary material for: Ancient medicinal plant rosemary contains a highly efficacious and isoform-selective KCNQ potassium channel opener
Source: Commun Biol. 2023 Jun 15;6:644. doi: 10.1038/s42003-023-05021-8 (PMC10272180; doi:10.1038/s42003-023-05021-8)
Supplement: Supplementary file 2 — Supplementary Information [file 42003_2023_5021_MOESM2_ESM.pdf]

Supplementary Data

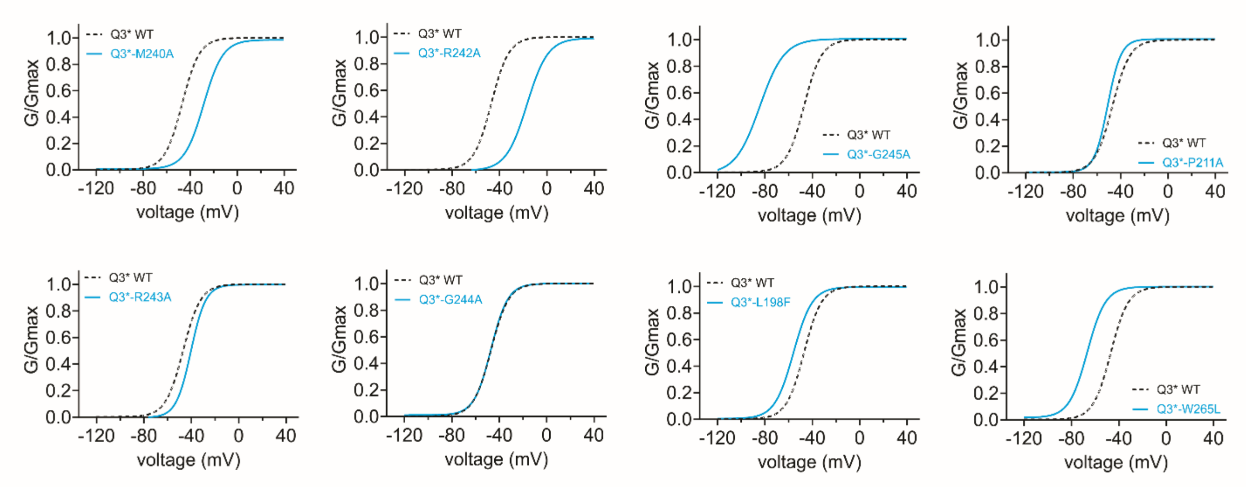

Supplementary Figure 1.

Mean baseline voltage dependence of wild-type (black; *n* = 60) versus mutant (cyan; n = 4-10) KCNQ3* channels as studied in Figure 8.

Supplementary Tables

Supplementary Table 1

KCNQ1	$V_{0.5}$ non-normalized tail current (mV)	$V_{0.5}$ Normalized tail current (mV)	Slope (mV)
Control	-35.85 ± 2.62	-36.82 ± 0.75	8.924 ± 0.72
1:100 Rosemary Ariel Parts	-38.25 ± 4.13 (*p*=0.6326; n=8)	-39.54 ± 0.82 (*p*=0.0283; n=8)	9.33 ± 0.82 (*p*=0.7139; n=8)

Statistics versus KCNQ1 in absence of rosemary ariel parts. Values indicate mean ± SEM.

KCNQ2	$V_{0.5}$ non-normalized tail current (mV)	$V_{0.5}$ Normalized tail current (mV)	Slope (mV)
Control	-45.37 ± 8.62	-43.61 ± 1.70	6.86 ± 1.74

1:100 Rosemary Ariel Parts	-53.56 ± 8.77 (*p*=0.5205; n=6)	-51.27 ± 1.76 (*p*=0.0107; n=6)	7.74 ± 1.19 (*p*=0.6863; n=6)

Statistics versus KCNQ2 in absence of rosemary ariel parts. Values indicate mean ± SEM.

**KCNQ3***	**$V_{0.5}$ non-normalized tail current (mV)**	**$V_{0.5}$ Normalized tail current (mV)**	**Slope (mV)**
Control	-48.24 ± 6.19	-48.76 ± 1.30	5.21 ± 0.66
1:100 Rosemary Ariel Parts	-57.00 ± 3.93 (*p*=0.2646; n=6)	-57.96 ± 2.33 (*p*=0.0090; n=6)	3.72 ± 0.18 (*p*=0.0743; n=6)

Statistics versus KCNQ3* in absence of rosemary ariel parts. Values indicate mean ± SEM.

Supplementary Table 2

**KCNQ2/3**	**$V_{0.5}$ non-normalized tail current (mV)**	**$V_{0.5}$ Normalized tail current (mV)**	**Slope (mV)**
Control	-43.81 ± 3.39	-44.41 ± 0.47	5.30 ± 0.44
1:100 Rosemary Ariel Parts	-51.82 ± 3.75 (*p*=0.1289; n=11)	-53.43 ± 0.71 (<0.0001; n=11)	6.09 ± 0.47 (*p*=0.2341; n=11)

Statistics versus KCNQ2/3 in absence of rosemary ariel parts. Values indicate mean ± SEM.

**KCNQ2/3**	**$V_{0.5}$ non-normalized tail current (mV)**	**$V_{0.5}$ Normalized tail current (mV)**	**Slope (mV)**
Control	-39.53 ± 3.53	-39.14 ± 0.74	4.80 ± 2.06
1:100 Rosemary Flower	-46.59 ± 5.03 (*p*=0.2875; n=5)	-46.42 ± 1.35 (*p*=0.0030; n=5)	5.92 ± 0.94 (*p*=0.6397; n=5)

Statistics versus KCNQ2/3 in absence of rosemary flower. Values indicate mean ± SEM.

KCNQ2/3	$V_{0.5}$ non-normalized tail current (mV)	$V_{0.5}$ Normalized tail current (mV)	Slope (mV)
Control	-33.04 ± 7.45	-32.30 ± 0.97	6.15 ± 0.60
1:100 Rosemary Stem	-40.14 ± 5.76 (*p*=0.4693; n=6)	-38.88 ± 1.06 (*p*=0.0009; n=6)	6.54 ± 1.53 (*p*=0.8197; n=6)

Statistics versus KCNQ2/3 in absence of rosemary stem. Values indicate mean ± SEM.

KCNQ3/5	$V_{0.5}$ non-normalized tail current (mV)	$V_{0.5}$ Normalized tail current (mV)	Slope (mV)
Control	-43.79 ± 2.82	-44.61 ± 0.43	7.85 ± 0.38
1:100 Rosemary Ariel Parts	-72.15 ± 3.98 (*p*=0.0008; n=5)	-72.15 ± 2.26 (*p*=0.0002; n=5)	612.12 ± 1.93 (*p*=0.0908; n=5)

Statistics versus KCNQ3/5 in absence of rosemary ariel parts. Values indicate mean ± SEM.

Supplementary Table 3

KCNQ3*	$V_{0.5}$ non-normalized tail current (mV)	$V_{0.5}$ Normalized tail current (mV)	Slope (mV)
Control	-49.07 ± 2.89	-50.95 ± 0.78	5.64 ± 0.70
100 µM Carnosic acid	-111.1 ± 5.75 (<0.0001; n=5)	-103.6 ± 0.62 (<0.0001; n=5)	9.91 ± 0.62 (*p*=0.0019; n=5)

Statistics versus KCNQ3* in absence of carnosic acid. Values indicate mean ± SEM.

KCNQ3*	$V_{0.5}$ non-normalized tail current (mV)	$V_{0.5}$ Normalized tail current (mV)	Slope (mV)
Control	-50.94 ± 3.99	-50.90 ± 1.49	5.67 ± 1.31
100 µM Homoplantaginin	-55.66 ± 4.03 (*p*=0.4294; n=5)	-57.70 ± 1.18 (*p*=0.0078; n=5)	5.51 ± 0.99 (*p*=0.9249; n=5)

Statistics versus KCNQ3* in absence of homoplantaginin. Values indicate mean ± SEM.

KCNQ3*	$V_{0.5}$ non-normalized tail current (mV)	$V_{0.5}$ Normalized tail current (mV)	Slope (mV)
Control	-44.79 ± 2.49	-46.39 ± 2.12	3.19 ± 1.44
100 µM Quinic acid	-51.87 ± 2.53 (*p*=0.0812; n=5)	-53.04 ± 1.09 (*p*=0.0317; n=5)	3.96 ± 0.88 (*p*=0.6628; n=5)

Statistics versus KCNQ3* in absence of quinic acid. Values indicate mean ± SEM.

KCNQ3*	$V_{0.5}$ non-normalized tail current (mV)	$V_{0.5}$ Normalized tail current (mV)	Slope (mV)
Control	-52.36 ± 3.06	-53.07 ± 0.72	4.75 ± 0.60
100 µM Rosmarinic acid	-50.09 ± 3.47 (*p*=0.6498; n=3)	-49.64 ± 0.84 (*p*=0.0374; n=3)	4.36 ± 0.60 (*p*=0.7245; n=3)

Statistics versus KCNQ3* in absence of rosmarinic acid. Values indicate mean ± SEM.

KCNQ3*	$V_{0.5}$ non-normalized tail current (mV)	$V_{0.5}$ Normalized tail current (mV)	Slope (mV)
Control	-46.21 ± 4.39	-45.21 ± 1.00	4.82 ± 0.80
100 µM Syringic acid	-51.58 ± 5.36 (*p*=0.4614; n=5)	-49.16 ± 1.21 (*p*=0.0370; n=5)	4.92 ± 1.03 (*p*=0.9409; n=5)

Statistics versus KCNQ3* in absence of syringic acid. Values indicate mean ± SEM.

KCNQ3*	$V_{0.5}$ non-normalized tail current (mV)	$V_{0.5}$ Normalized tail current (mV)	Slope (mV)
Control	-49.32 ± 2.95	-48.37 ± 0.72	4.88 ± 0.65
100 µM Ursolic acid	-52.94 ± 3.12 (*p*=0.4238; n=5)	-52.27 ± 0.61 (*p*=0.0035; n=5)	4.49 ± 0.53 (*p*=0.6548; n=5)

Statistics versus KCNQ3* in absence of ursolic acid. Values indicate mean ± SEM.

KCNQ3*	$V_{0.5}$ non-normalized tail current (mV)	$V_{0.5}$ Normalized tail current (mV)	Slope (mV)
Control	-45.54 ± 1.75	-45.50 ± 0.54	4.52 ± 0.42
30 µM Hesperidin	-55.83 ± 1.97 (*p*=0.0046; n=5)	-55.97 ± 0.57 (<0.0001; n=5)	4.65 ± 0.46 (*p*=0.8399; n=5)

Statistics versus KCNQ3* in absence of hesperidin. Values indicate mean ± SEM.

Supplementary Table 4

KCNQ3*	$V_{0.5}$ Normalized tail current (mV)
Control	-51.77 ± 0.69
0.1 µM Carnosic acid	-55.21 ± 0.45 (*p*=0.2200; n=5)
1 µM Carnosic acid	-59.60 ± 0.47 (*p*=0.0005; n=5)
3 µM Carnosic acid	-63.92 ± 0.74 (<0.0001; n=5)
10 µM Carnosic acid	-73.28 ± 1.41 (<0.0001; n=5)
30 µM Carnosic acid	-92.58 ± 2.21 (<0.0001; n=5)
100 µM Carnosic acid	-104.6 ± 1.44 (<0.0001; n=5)

Statistics versus KCNQ3* in absence of carnosic acid. Values indicate mean ± SEM.

KCNQ3*	EC_{50} Fold change -60 mV	EC_{50} $V_{0.5}$ Activation (mV)	EC_{50} E_M (mV)
	5.43 ± 0.37 µM (n=5)	18 ± 0.72 µM (n=5)	1.19 ± 0.19 µM (n=5)

Data versus KCNQ3* in absence of carnosic acid. Values indicate mean ± SEM.

**KCNQ2**	**$V_{0.5}$ Normalized tail current (mV)**
Control	-36.97 ± 1.66
0.1 µM Carnosic acid	-41.03 ± 1.43 (*p*=0.0583; n=5)
1 µM Carnosic acid	-44.14 ± 0.95 (*p*=0.0004; n=5)
3 µM Carnosic acid	-46.74 ± 0.85 (<0.0001; n=5)
10 µM Carnosic acid	-45.67 ± 0.73 (<0.0001; n=5)
30 µM Carnosic acid	-45.20 ± 0.88 (<0.0001; n=5)
100 µM Carnosic acid	-43.10 ± 0.64 (*p*=0.0021; n=5)

Statistics versus KCNQ2 in absence of carnosic acid. Values indicate mean ± SEM.

**KCNQ2/3**	**$V_{0.5}$ Normalized tail current (mV)**
Control	-41.31 ± 0.92
0.1 µM Carnosic acid	-44.63 ± 0.46 (*p*=0.0028; n=5)
1 µM Carnosic acid	-45.44 ± 0.41 (*p*=0.0002; n=5)
3 µM Carnosic acid	-46.40 ± 0.45 (<0.0001; n=5)
10 µM Carnosic acid	-47.94 ± 0.61 (<0.0001; n=5)
30 µM Carnosic acid	-49.09 ± 0.55 (<0.0001; n=5)
100 µM Carnosic acid	-49.95 ± 0.65 (<0.0001; n=5)

Statistics versus KCNQ2/3 in absence of carnosic acid. Values indicate mean ± SEM.

Supplementary Table 5

KCNQ3*	$V_{0.5}$ Normalized tail current (mV)	Slope (mV)
Control	-43.64 ± 0.43	4.36 ± 0.28
5 µM Carnosic acid	-63.93 ± 1.08 (<0.0001; n=9)	7.61 ± 0.94 (*p*=0.0087; n=9)

Statistics versus KCNQ3* in absence of carnosic acid. Values indicate mean ± SEM.

KCNQ3*	$V_{0.5}$ Normalized tail current (mV)	Slope (mV)
Control	-32.23 ± 0.28	4.55 ± 0.25
5 µM Carnosic acid (Post 3 hrs. incubation 30 µM Wortmannin)	-46.82 ± 0.64 (<0.0001; n=5)	6.70 ± 0.56 (*p*=0.0145; n=5)

Statistics versus KCNQ3* in absence of carnosic acid. Values indicate mean ± SEM.

Supplementary Table 6

KCNQ2/3	$V_{0.5}$ non-normalized tail current (mV)	$V_{0.5}$ Normalized tail current (mV)	Slope (mV)
Control	-37.91 ± 2.41	-37.50 ± 0.51	6.95 ± 0.45
30 µM Hesperidin	-48.30 ± 2.77 (*p*=0.0226; n=5)	-48.08 ± 0.71 (<0.0001; n=5)	7.73 ± 0.60 (*p*=0.3310; n=5)

Statistics versus KCNQ2/3 in absence of hesperidin. Values indicate mean ± SEM.

KCNQ2/3	$V_{0.5}$ non-normalized tail current (mV)	$V_{0.5}$ Normalized tail current (mV)	Slope (mV)
Control	-45.69 ± 2.31	-46.69 ± 0.97	5.57 ± 0.72
100 µM Homoplantaginin	-45.32 ± 1.91 (*p*=0.9049; n=5)	-46.83 ± 0.95 (*p*=0.9204; n=5)	5.78 ± 0.93 (*p*=0.8630; n=5)

Statistics versus KCNQ2/3 in absence of homoplantaginin. Values indicate mean ± SEM.

KCNQ2/3	$V_{0.5}$ non-normalized tail current (mV)	$V_{0.5}$ Normalized tail current (mV)	Slope (mV)
Control	-36.67 ± 2.80	-36.37 ± 1.65	7.93 ± 1.45
100 µM Ursolic acid	-40.64 ± 1.83 (*p*=0.2746; n=5)	-40.90 ± 0.77 (*p*=0.0497; n=5)	6.36 ± 0.67 (*p*=0.3660; n=5)

Statistics versus KCNQ2/3 in absence of ursolic acid. Values indicate mean ± SEM.

KCNQ2/3	$V_{0.5}$ non-normalized tail current (mV)	$V_{0.5}$ Normalized tail current (mV)	Slope (mV)
Control	-46.58 ± 1.42	-48.43 ± 0.73	4.43 ± 0.68
100 µM Quinic acid	-45.78 ± 1.64 (*p*=0.7221; n=5)	-47.87 ± 0.94 (*p*=0.6513; n=5)	4.07 ± 0.85 (*p*=0.7498; n=5)

Statistics versus KCNQ2/3 in absence of quinic acid. Values indicate mean ± SEM.

KCNQ2/3	$V_{0.5}$ non-normalized tail current (mV)	$V_{0.5}$ Normalized tail current (mV)	Slope (mV)
Control	-49.32 ± 2.69	-50.00 ± 1.07	5.67 ± 0.94
100 µM Rosmarinic acid	-48.04 ± 1.86 (*p*=0.7070; n=5)	-49.12 ± 1.11 (*p*=0.5838; n=5)	5.15 ± 1.00 (*p*=0.7147; n=5)

Statistics versus KCNQ2/3 in absence of rosmarinic acid. Values indicate mean ± SEM.

KCNQ2/3	$V_{0.5}$ non-normalized tail current (mV)	$V_{0.5}$ Normalized tail current (mV)	Slope (mV)
Control	-44.66 ± 1.80	-46.07 ± 0.49	5.54 ± 0.41
100 µM Syringic acid	-44.21 ± 2.07 (*p*=0.8738; n=5)	-47.76 ± 0.91 (*p*=0.1520; n=5)	5.13 ± 0.75 (*p*=0.6479; n=5)

Statistics versus KCNQ2/3 in absence of syringic acid. Values indicate mean ± SEM.

KCNQ2	$V_{0.5}$ Normalized tail current (mV)	Slope (mV)
Control	-46.17 ± 0.41	6.21 ± 0.35
100 µM Hesperidin	-45.73 ± 0.48 (*p*=0.5060; n=5)	6.54 ± 0.41 (*p*=0.5578; n=5)

Statistics versus KCNQ2 in absence of hesperidin Values indicate mean ± SEM.

Supplementary Table 7

KCNQ5	$V_{0.5}$ Normalized tail current (mV)
Control	-43.83 ± 0.34
0.1 µM Carnosic acid	-42.57 ± 0.34 (*p*=0.0926; n=5)
1 µM Carnosic acid	-42.20 ± 0.27 (*p*=0.0184; n=5)
3 µM Carnosic acid	-42.54 ± 0.31 (*p*=0.0820; n=5)
10 µM Carnosic acid	-43.64 ± 0.36 (*p*=0.9979; n=5)
30 µM Carnosic acid	-45.91 ± 0.40 (*p*=0.0020; n=5)
100 µM Carnosic acid	-50.99 ± 0.49 (<0.0001; n=5)

Statistics versus KCNQ5 in absence of carnosic acid. Values indicate mean ± SEM.

KCNQ3/5	$V_{0.5}$ Normalized tail current (mV)
Control	-36.80 ± 0.38
0.1 µM Carnosic acid	-41.88 ± 0.50 (<0.0001; n=5)
1 µM Carnosic acid	-47.59 ± 0.51 (<0.0001; n=5)
3 µM Carnosic acid	-52.07 ± 0.47 (<0.0001; n=5)
10 µM Carnosic acid	-56.02 ± 0.57 (<0.0001; n=5)
30 µM Carnosic acid	-62.10 ± 0.87 (<0.0001; n=5)
100 µM Carnosic acid	-68.83 ± 1.11 (<0.0001; n=5)

Statistics versus KCNQ3/5 in absence of carnosic acid. Values indicate mean ± SEM.

**KCNQ2/5**	**$V_{0.5}$ Normalized tail current (mV)**
Control	-34.07 ± 1.08
0.1 µM Carnosic acid	-40.58 ± 1.11 (<0.0001; n=5)
1 µM Carnosic acid	-44.54 ± 0.73 (<0.0001; n=5)
3 µM Carnosic acid	-47.07 ± 0.55 (<0.0001; n=5)
10 µM Carnosic acid	-46.44 ± 0.68 (<0.0001; n=5)
30 µM Carnosic acid	-45.89 ± 0.76 (<0.0001; n=5)
100 µM Carnosic acid	-46.50 ± 0.97 (<0.0001; n=5)

Statistics versus KCNQ2/5 in absence of carnosic acid. Values indicate mean ± SEM.

**KCNQ2/3/5**	**$V_{0.5}$ Normalized tail current (mV)**
Control	-38.98 ± 1.02
0.1 µM Carnosic acid	-43.51 ± 1.17 (*p*=0.0122; n=5)
1 µM Carnosic acid	-47.43 ± 0.94 (<0.0001; n=5)
3 µM Carnosic acid	-49.89 ± 0.88 (<0.0001; n=5)
10 µM Carnosic acid	-52.31 ± 0.77 (<0.0001; n=5)
30 µM Carnosic acid	-54.68 ± 0.89 (<0.0001; n=5)
100 µM Carnosic acid	-57.42 ± 1.03 (<0.0001; n=5)

Statistics versus KCNQ2/3/5 in absence of carnosic acid. Values indicate mean ± SEM.

	**EC_{50} $V_{0.5}$ Activation (mV)**	**EC_{50} E_M (mV)**
KCNQ5	231 ± 75 µM (n=5)	43.4 ± 0.28 µM (n=5)
KCNQ3/5	4.53 ± 0.11 µM (n=5)	4.21 ± 0.16 µM (n=5)
KCNQ2/5	0.74 ± 0.12 µM (n=5)	4.76 ± 0.34 µM (n=5)
KCNQ2/3/5	2.02 ± 0.44 µM (n=5)	3.13 ± 0.44 µM (n=5)

EC_{50} values for KCNQ5-containing complexes with carnosic acid. Values indicate mean ± SEM.

Supplementary Table 8

KCNQ3*	$V_{0.5}$ Normalized tail current (mV)	Slope (mV)
Control	-41.36 ± 1.01	7.89 ± 0.88
5 µM Aloperine	-46.72 ± 0.78 (*p*=0.0016; n=5)	6.96 ± 0.67 (*p*=0.6191; n=5)
5 µM Aloperine + 5 µM Carnosic acid	-52.53 ± 0.77 (<0.0001; n=5)	8.20 ± 0.67 (*p*=0.9440; n=5)

Statistics versus KCNQ3* in absence of aloperine and Aloperine + carnosic acid. Values indicate mean ± SEM.

KCNQ3*-M240A	$V_{0.5}$ Normalized tail current (mV)	Slope (mV)
Control	-28.62 ± 0.73	8.18 ± 0.64
100 µM Carnosic acid	-66.81 ± 1.80 (<0.0001; n=6)	17.31 ± 1.56 (*p*=0.0012; n=6)

Statistics versus KCNQ3* in absence of carnosic acid. Values indicate mean ± SEM.

KCNQ3*-R242A	$V_{0.5}$ Normalized tail current (mV)	Slope (mV)
Control	-19.15 ± 0.75	8.18 ± 0.64
100 µM Carnosic acid	-42.88 ± 0.68 (<0.0001; n=10)	8.88 ± 0.59 (*p*=0.1055; n=10)

Statistics versus KCNQ3* in absence of carnosic acid. Values indicate mean ± SEM.

KCNQ3*-R243A	$V_{0.5}$ Normalized tail current (mV)	Slope (mV)
Control	-40.79 ± 0.39	5.87 ± 0.35
100 µM Carnosic acid	-64.93 ± 0.61 (<0.0001; n=6)	9.48 ± 0.54 (*p*=0.0004; n=6)

Statistics versus KCNQ3* in absence of carnosic acid. Values indicate mean ± SEM.

KCNQ3*-G244A	$V_{0.5}$ Normalized tail current (mV)	Slope (mV)
Control	-47.62 ± 1.02	6.65 ± 0.89
100 µM Carnosic acid	-97.68 ± 3.18 (<0.0001; n=5)	11.55 ± 1.94 (*p*=0.0644; n=5)

Statistics versus KCNQ3* in absence of carnosic acid. Values indicate mean ± SEM.

KCNQ3*-G245A	$V_{0.5}$ Normalized tail current (mV)	Slope (mV)
Control	-84.16 ± 1.58	10.33 ± 1.40
100 µM Carnosic acid	-129.4 ± 1.24 (<0.0001; n=5)	12.79 ± 4.65 (*p*=0.6352; n=5)

Statistics versus KCNQ3* in absence of carnosic acid. Values indicate mean ± SEM.

KCNQ3*-R242A	$V_{0.5}$ Normalized tail current (mV)
Control	-16.97 ± 1.08
0.1 µM Carnosic acid	-21.11 ± 0.68 (*p*=0.0019; n=5)
1 µM Carnosic acid	-24.52 ± 0.42 (<0.0001; n=5)
3 µM Carnosic acid	-27.59 ± 0.38 (<0.0001; n=5)
10 µM Carnosic acid	-30.42 ± 0.37 (<0.0001; n=5)
30 µM Carnosic acid	-35.30 ± 0.41 (<0.0001; n=5)
100 µM Carnosic acid	-41.76 ± 0.78 (<0.0001; n=5)

Statistics versus KCNQ3*-R242A in absence of carnosic acid. Values indicate mean ± SEM.

Supplementary Table 9

KCNQ3*-W265L	$V_{0.5}$ Normalized tail current (mV)
Control	-68.38 ± 0.86
0.1 µM Carnosic acid	-75.60 ± 1.02 (*p*=0.0133; n=5)
1 µM Carnosic acid	-86.19 ± 1.09 (<0.0001; n=5)
3 µM Carnosic acid	-96.66 ± 1.64 (<0.0001; n=5)
10 µM Carnosic acid	-105.3 ± 2.56 (<0.0001; n=5)
30 µM Carnosic acid	-125.0 ± 1.87 (<0.0001; n=5)
100 µM Carnosic acid	-146.0 ± 1.92 (<0.0001; n=5)

Statistics versus KCNQ3*-W265L in absence of carnosic acid. Values indicate mean ± SEM.

KCNQ3*-P211A	$V_{0.5}$ Normalized tail current (mV)
Control	-47.89 ± 1.50
0.1 µM Carnosic acid	-55.16 ± 1.00 (*p*=0.2064; n=5)
1 µM Carnosic acid	-59.20 ± 1.17 (*p*=0.0185; n=5)
3 µM Carnosic acid	-61.55 ± 1.44 (*p*=0.0036; n=5)
10 µM Carnosic acid	-78.35 ± 1.93 (<0.0001; n=5)
30 µM Carnosic acid	-98.23 ± 4.44 (<0.0001; n=5)
100 µM Carnosic acid	-110.7 ± 3.83 (<0.0001; n=5)

Statistics versus KCNQ3*-P211A in absence of carnosic acid. Values indicate mean ± SEM.

**KCNQ3*-L198F**	**$V_{0.5}$ Normalized tail current (mV)**
Control	-55.88 ± 1.11
0.1 µM Carnosic acid	-58.22 ± 0.59 (*p*=0.9404; *n*=4)
1 µM Carnosic acid	-62.90 ± 0.79 (*p*=0.1405; *n*=4)
3 µM Carnosic acid	-73.56 ± 0.79 (<0.0001; *n*=4)
10 µM Carnosic acid	-91.66 ± 1.99 (<0.0001; *n*=4)
30 µM Carnosic acid	-116.6 ± 3.44 (<0.0001; *n*=4)
100 µM Carnosic acid	-136.6 ± 3.98 (<0.0001; *n*=4)

Statistics versus KCNQ3*-L198F in absence of carnosic acid. Values indicate mean ± SEM.

	**EC_{50} $V_{0.5}$ Activation (mV)**	**EC_{50} E_M (mV)**
KCNQ3*-R242A	8.31 ± 0.14 µM (n=5)	n.a.
KCNQ3*-W265L	2.11 ± 0.09 µM (n=5)	0.14 ± 0.03 µM (n=5)
KCNQ3*-P211A	15.25 ± 0.07 µM (n=5)	3.21 ± 0.21 µM (n=5)
KCNQ3*-L198F	6.28 ± 0.08 µM (n=5)	1.15 ± 0.12 µM (n=5)

Supplementary Table 10

**KCNQ2**	**$V_{0.5}$ Normalized tail current (mV)**	**Slope (mV)**
Control	-49.97 ± 0.88	3.86 ± 0.99
100 µM Carnosol	-57.08 ± 1.31 (*p*=0.0057; n=4)	3.97 ± 1.31 (*p*=0.9489; n=4)

Statistics versus KCNQ2 in absence of carnosol. Values indicate mean ± SEM.

KCNQ2/3	$V_{0.5}$ Normalized tail current (mV)	Slope (mV)
Control	-48.27 ± 0.57	6.06 ± 0.49
100 µM Carnosol	-47.80 ± 0.87 (*p*=0.7256; n=5)	5.82 ± 0.75 (*p*=0.7966; n=5)

Statistics versus KCNQ2/3 in absence of carnosol. Values indicate mean ± SEM.

KCNQ3*	$V_{0.5}$ Normalized tail current (mV)	Slope (mV)
Control	-49.98 ± 0.68	4.45 ± 0.67
100 µM Carnosol	-61.13 ± 1.28 <0.0001; *n*=10)	5.00 ± 1.11 (*p*=0.6775; *n*=10)

Statistics versus KCNQ3* in absence of carnosol. Values indicate mean ± SEM.

KCNQ2/3	$V_{0.5}$ Normalized tail current (mV)	Slope (mV)
Control	-34.59 ± 0.58	5.85 ± 0.49
100 µM Dimethylcarnosol	-37.81 ± 0.73 (*p*=0.0030; *n*=10)	6.71 ± 0.64 (*p*=0.3010; *n*=10)

Statistics versus KCNQ2/3 in absence of dimethylcarnosol. Values indicate mean ± SEM.

KCNQ3*	$V_{0.5}$ Normalized tail current (mV)	Slope (mV)
Control	-43.05 ± 0.40	4.27 ± 0.33
100 µM Dimethylcarnosol	-49.25 ± 0.43 (<0.0001; *n*=10)	4.92 ± 0.40 (*p*=0.2266; *n*=10)

Statistics versus KCNQ3* in absence of dimethylcarnosol. Values indicate mean ± SEM.

KCNQ2	$V_{0.5}$ Normalized tail current (mV)	Slope (mV)
Control	-47.49 ± 0.53	6.76 ± 0.46
10 µM Carnosic gamma lactone	-46.18 ± 0.46 (*p*=0.0996; n=5)	6.47 ± 0.40 (*p*=0.6472; n=5)

Statistics versus KCNQ2 in absence of carnosic gamma lactone. Values indicate mean ± SEM.

KCNQ2/3	$V_{0.5}$ Normalized tail current (mV)	Slope (mV)
Control	-32.40 ± 0.50	6.57 ± 0.44
10 µM Carnosic gamma lactone	-40.71 ± 0.71 (<0.0001; *n*=10)	6.78 ± 0.62 (*p*=0.7859; *n*=10)

Statistics versus KCNQ2/3 in absence of carnosic gamma lactone. Values indicate mean ± SEM.

**KCNQ3***	**$V_{0.5}$ Normalized tail current (mV)**	**Slope (mV)**
Control	-45.30 ± 0.84	4.60 ± 0.66
10 µM Carnosic gamma lactone	-54.06 ± 0.70 (<0.0001; *n*=9)	4.81 ± 0.56 (*p*=0.8115; *n*=9)

Statistics versus KCNQ3* in absence of carnosic gamma lactone. Values indicate mean ± SEM.

**KCNQ2/3**	**$V_{0.5}$ Normalized tail current (mV)**	**Slope (mV)**
Control	-33.18 ± 0.67	8.03 ± 0.59
10 µM Methyl carnosate	-34.00 ± 0.97 (*p*=0.4967; *n*=10)	10.46 ± 0.87 (*p*=0.0346; *n*=10)

Statistics versus KCNQ2/3 in absence of methyl carnosate. Values indicate mean ± SEM.

**KCNQ3***	**$V_{0.5}$ Normalized tail current (mV)**	**Slope (mV)**
Control	-41.39 ± 0.71	3.44 ± 0.78
10 µM Methyl carnosate	-47.09 ± 0.74 (*p*=0.0005; n=5)	4.63 ± 0.74 (*p*=0.3006; n=5)

Statistics versus KCNQ3* in absence of methyl carnosate. Values indicate mean ± SEM.

**KCNQ3***	**$V_{0.5}$ Normalized tail current (mV)**	**Slope (mV)**
Control	-43.88 ± 0.67	4.80 ± 0.55
100 µM Pisiferic acid	-51.70 ± 0.51 (<0.0001; *n*=10)	4.89 ± 0.45 (*p*=0.9007; *n*=10)

Statistics versus KCNQ3* in absence of pisiferic acid. Values indicate mean ± SEM.

**KCNQ2/3**	**$V_{0.5}$ Normalized tail current (mV)**	**Slope (mV)**
Control	-34.76 ± 0.46	6.55 ± 0.40
100 µM Pisiferic acid	-42.05 ± 0.63 (<0.0001; *n*=10)	7.44 ± 0.55 (*p*=0.2086; *n*=10)

Statistics versus KCNQ2/3 in absence of pisiferic acid. Values indicate mean ± SEM.

**KCNQ2/3**	**$V_{0.5}$ Normalized tail current (mV)**	**Slope (mV)**
Control	-33.65 ± 0.40	6.11 ± 0.35
10 µM Carnosic acid diacetate	-38.43 ± 0.56 (<0.0001; *n*=10)	6.11 ± 0.49 (>0.9999; *n*=10)

Statistics versus KCNQ2/3 in absence of carnosic diacetate. Values indicate mean ± SEM.

KCNQ3*	$V_{0.5}$ Normalized tail current (mV)	Slope (mV)
Control	-46.89 ± 0.60	5.38 ± 0.51
10 µM Carnosic acid diacetate	-50.38 ± 0.69 (*p*=0.0013; *n*=10)	5.06 ± 0.63 (*p*=0.6978; *n*=10)

Statistics versus KCNQ3* in absence of carnosic diacetate. Values indicate mean ± SEM.
